# Cross sectional analysis of student-led surgical societies in fostering medical student interest in Canada

**DOI:** 10.1186/s12909-019-1502-5

**Published:** 2019-03-08

**Authors:** Jin Soo A. Song, Connor McGuire, Michael Vaculik, Alexander Morzycki, Madelaine Plourde

**Affiliations:** 1 Faculty of Medicine, Dalhousie University, Halifax, Nova Scotia Canada; 2 Division of Thoracic Surgery, Dalhousie University, Halifax, Nova Scotia Canada; 3 Dalhousie Medical School, 5850 College St, Halifax, Nova Scotia B3H 1X5 Canada

**Keywords:** Medical education, Competence, And mentors

## Abstract

**Background:**

The objective of this study was to examine how surgery interest groups (SIGs) across Canada function and influence medical students’ interest in surgical careers.

**Methods:**

Two unique surveys were distributed using a cross sectional design. The first was sent to SIG executives and the second to SIG members enrolled at a Canadian medical school in the 2016/17 academic year. The prior focused on the types of events hosted, SIG structure/ supports, and barriers/ plans for improvement. The second questionnaire focused on student experience, involvement, and suggestions for improvement.

**Results:**

SIG executives became involved in SIG through classmates and colleagues (8/17, 47%). Their roles focused on organizing events (17/17, 100%), facilitating student contact with resident/surgeons (17/17, 100%), and organizing funding (13/17, 76%). Surgical skills events were among the most successful and well received by students (15/17, 88%). Major barriers faced by SIG executives during their tenure included time conflicts with other interest groups (13/17, 76%), lack of funding (8/17, 47%), and difficulty booking spaces for events (8,17, 47%). SIGs were found to facilitate improvement in basic surgical skills (*μ* = 3.89/5 ± 0.70) in a comfortable environment (*μ* = 4.02/5, ±0.6), but were not helpful with final block examinations (*μ* = 2.98/5, ±0.80). Members indicated that more skills sessions, panel discussion and shadowing opportunities would be beneficial additions. Overall, members felt that SIGs increased their interest in surgical careers (*μ* = 3.50/5, ±0.79).

**Conclusion:**

Canadian SIGs not only play a critical role in early exposure, but may provide a foundation to contribute to student success in surgery.

**Electronic supplementary material:**

The online version of this article (10.1186/s12909-019-1502-5) contains supplementary material, which is available to authorized users.

## Background

In recent decades, there has been increasing discrepancy between the growing demand for surgeons and the number of graduating medical students entering training programs in surgical disciplines. Current literature estimates the number of Canadian medical students choosing a surgical discipline as their first choice residency position has decreased from 24.7 to 21.7% between 1998 and 2006 with similar trends in the United States (US) [1–5]. Studies estimate that by 2028 there will be an additional 18% nationwide shortfall of general surgeons in the US [6–8]. This has led to a growing interest in the rationale for why incoming medical students are drawing away from surgery, and how this phenomenon can be mitigated and reversed.

When considering a future medical career, common considerations include role modeling, personality fit, lifestyle, and debt load [9]. Amongst the myriad of variables, work life balance and lifestyle concerns are persistently the most commonly cited [4, 6, 9, 10]. Due to undergraduate medical programs increasingly focusing on primary care specialities such as family medicine, anatomy and pathology content in medical schools have been markedly reduced [11]. Hence, as the vast majority of medical students receive limited to no exposure to surgical specialties prior to their 3rd year of medical school or clerkship, students often rely on anecdotes and discussions with classmates to gain insight into a career as a surgeon [12]. In addition, the importance of clarifying ideals surrounding lifestyle and work life balance in surgery is paramount to encourage student interest [4, 6, 13–15].

Surgery interest groups (SIGs) are student led, academically supported and institution specific groups that aim to bolster interest in surgery through a variety of events, from academic lecture series, to technical skills practice such as suturing and knot tying. SIGs are found across the world, however are most prominently studied in North America [5, 6, 13]. In addition, they provide important opportunities to build and foster relationships with residents and faculty and facilitate research involvement. Previous studies demonstrate that SIGs have the ability to fortify interest and increase enrollment in surgical residency programs [5, 6, 14]. Therefore, evaluation of how these SIGs operate and consequently influence medical students is an essential endeavor. The objective of our study is to petition SIG leaders and members across the country, to elucidate a comprehensive perspective on how these organizations function and influence medical students.

## Methods

Two online surveys were distributed via Opinio software (ObjectPlant, Inc., Oslo, Norway) (Additional files 1 and 2). The first, referred to the executive survey, was distributed to the previous executives of surgery interest groups at 13 participating Canadian medical schools. Executives were medical students who were in charge of leading their respective schools SIG. The second survey, referred to as the member survey, was distributed to students in all 4 years of study enrolled at a Canadian medical school in the 2016/2017 academic year who took part in their respective school’s SIG. The surveys were developed from an extensive literature review and following discussion and input from several medical students and staff members. Informed consent was considered obtained if respondents completed the entirety of the survey, which was detailed at the beginning of the survey. Consent detailed that participation was entirely voluntary and participants could withdraw at any time with no repercusions.

The executive survey consisted of 23 questions concerning previous executives experience with SIG, including the types of events they hosted, the structure of their interest group, support and resources, as well as barriers and plans for improvement. The member survey consisted of 22 closed-ended questions. Respondents were asked to rank their agreement with each comment on a Likert scale from 1 (strongly disagree) to 5 (strongly agree). Questions assessed medical students’ experience with their local SIG, including which events they found most beneficial, how their involvement affected their interest and competencies, and suggestions for improvement.

Statistical analyses were done in SPSS Version 22. Graphics were created using Prism (GraphPad®, San Diego, CA). Univariate descriptive analysis were performed for all variables. Free text responses were itemized and thematically analyzed. The data was reviewed and content categories were developed from responses, with definitions for each category. The data were coded by these categories and the number of responses in each category was summed. Approval for this study was obtained through the local research ethics board at the Nova Scotia Health Authority.

## Results

### Executive survey

Of a total of 22 previous leaders or executives, 17 participants from 10 Canadian academic institutions participated in the survey (76.9% participation rate) (Table 1). Leaders spent an average of 2.25 h per week (±1.39) operating their organization, with a yearly budget of 700 Canadian dollars (±576). Leaders most often became introduced to their societies through introduction by classmates (47%) or an interest group fair (41%), and all became executives due to their interest in surgery as a career (100%).

**Table 1:** Characteristics of participating SIG executives (*n* = 17)

Characteristics	Frequency	
	n	(%)
Overall	17	100
Executives Institution	7	75
Memorial University	1	8
Queen’s University	2	15
Northern University	3	23
University of Ottawa	2	15
University of Toronto	2	15
Université de Sherbrooke	1	8
University of Saskatchewan	1	8
University of Alberta	2	15
University of British Columbia	2	15

Their positions as executives were most frequently appointed through previous chairs (88%). Leadership roles included organizing events (100%), facilitating contact with surgeons/ residents (100%), organizing funding (76%), and skills teaching (71%). SIG leaders across the country organized a wide variety of events to facilitate student interest in surgery: surgeon Q&A/lifestyle nights (88%), surgical skills sessions (88%), resident Q&A/lifestyle night (71%), and lecture series (59%; Table 2). Events were predominantly led by medical students (47%) or surgical residents (29%). The most well attended events were surgical skills sessions (71%) and OR scrub sessions (12%). In terms of other programs SIGs felt they helped facilitate, promoting women in surgery (41%) and mentorship/observerships (29%) were most commonly described.

**Table 2:** Responses of participating SIG chairs (*n* = 17)

Response	Frequency	
	n	(%)
Initial introduction to SIG		
Friend/ Classmate	8	47
Interest Group fair	7	41
Email	2	12
Route to appointment	17	100
Appointed by previous chairs	15	88
Voted in by students	2	12
What made you first join the SIG?		
Interest in surgery as a career	17	100
Gain exposure/ experience in surgery	3	18
Getting more involved	2	12
To connect with residents/ staff	1	6
Help promote surgery	1	6
Improve medical education in surgery	1	6
Another position fell through	1	6
Staying updated	1	6
What were your roles as SIG executives		
Organize events	17	100
Contact surgeons/ residents	17	100
Organize funding	13	76
Student advocacy	11	65
Educational/ departmental	4	24
Junior medical student mentorship	5	29
Skills teaching	12	71
Organizing SEAD	4	24
Other	1	6
Did you believe you had sufficient funding to run planned events?		
Yes	12	71
No	5	29
What types of events were held throughout the year?		
Lecture series	10	59
Surgeon Q&A/ Surgeon lifestyle night	15	88
Resident Q&A/ Resident lifestyle night	12	71
Residency matching information night	9	53
Scrubbing in to operating room /sterile technique session	6	35
Surgical skills session	15	88
Career night	12	71
Full day events (Eg. ‘Surgery Saturdays’	5	29
Other	6	35
Who hosted the majority of events?		
Medical students	8	47
Staff surgeons	2	12
Residents	5	29
Other	2	12
Which event was the most successful/ best received?		
Surgical skills sessions	12	71
Scrubbing in to operating room /sterile technique session	2	12
Lecture series	1	6
Surgeon Q&A/ Surgeon lifestyle night	1	6
Which programs did SIG help facilitate?		
SEAD	4	24
Observerships	5	29
Transplant procurement	0	0
On-call shifts	1	6
Mentorship program (with residents)	5	29
Mentorship program (with staff)	0	0
Women in surgery	7	41
Other	1	6
Which advertising methods were used?		
E-mail	12	71
Posters	2	12
Class announcements	12	71
Interest group fairs	6	35
Flyers	0	0
Other (Facebook)	9	53
Which form of support did you receive?		
Financial	13	76
Administrative	8	47
Promotional	2	12
Staff surgeon liaison	10	59
Other	0	0
Was interest in your group maintained throughout the year?		
Strongly Agree	3	18
Agree	11	65
Neutral	3	18

The majority communicated through the dual modalities of email (71%) and Facebook (53%), as well as face-to-face class announcements (71%). For support, most organizations felt they received aid in the form of financial (76%), administrative (47%) and staff surgeon liaison (59%) assistance. The majority of SIGs (83%) agreed or strongly agreed that interest in their group was maintained throughout the year.

SIG executives faced numerous challenges during their tenure. The biggest barriers faced by groups included: Conflicts with other interest groups for time (76%), lack of funding (47%), and trouble booking space for events (47%; Table 3). The single biggest obstacle was conflicts with other interest groups for time (76%). The most commonly described aspects that need improvement were accessing more funding (24%) and collaboration with other interest groups/SIGs (18%). An equal proportion of SIG leaders felt that they were uncertain about the possibility of collaborating with SIG’s from other medical schools. The most commonly foreseen benefits to collaboration included increased access to surgeons (65%) and funding (53%) while the most commonly predicted pitfalls included more organizational hurdles (94%) and work (53%).

**Table 3:** Barriers faced by participating SIG chairs (*n* = 17)

Response	Frequency	
	n	(%)
What barriers did you face as chair?		
Financial	8	47
Trouble booking space for events	8	47
Poor resident involvement	3	18
Poor faculty involvement	2	12
Poor departmental/ university involvement	0	0
Poor student engagement	2	12
Conflicts with other interest groups for time	13	76
Trouble balancing medical school with running the surgery interest group	4	24
Other (Trouble receiving timely responses from staff surgeons regarding attendance)	1	6
What was the single biggest obstacle during your tenure?		
Financial	8	47
Poor faculty involvement	2	12
Poor student involvement	2	12
Poor resident involvement	3	18
Conflicts with other interest groups for time	13	76
Trouble getting space for events	8	47
Trouble balancing medical school and the surgery interest group	4	24
Other	3	18
How do you think your SIG could improve?		
More Funding	4	24
More guidance for events/ teaching skills	1	6
Hosting more events	2	12
Collaboration with other interest groups and SIGs nationally	3	18
More support from faculty	1	6
More resident involvement	1	6
More organizing	1	6
Delegate tasks for effectively	1	6
More chairs	1	6
More defined positions within the group	1	6
More space for events	1	6
Fever events, more higher yield events	1	6
Would you like to see a national, collaborative Canadian Surgery interest group?		
Unsure	8	47
Yes	8	47
No	1	6
In your opinion, what would be some of the benefits of having a national collaborative surgery interest group?		
Access to more funding	9	53
Standardizing events	8	47
Access/ connections to a greater number of surgeons	11	65
Access/ connections to a greater number of students	7	41
Other	0	0
In your opinion, what would be some of the pitfalls of a national surgery interest group be?		
More bureaucratic/ organizational hurdles	16	94
More work	9	53
It wouldn’t work	3	18
It wouldn’t improve our existing model	3	18
Less funding	0	0
Other	4	24

### Member survey

**Fig. 1:**
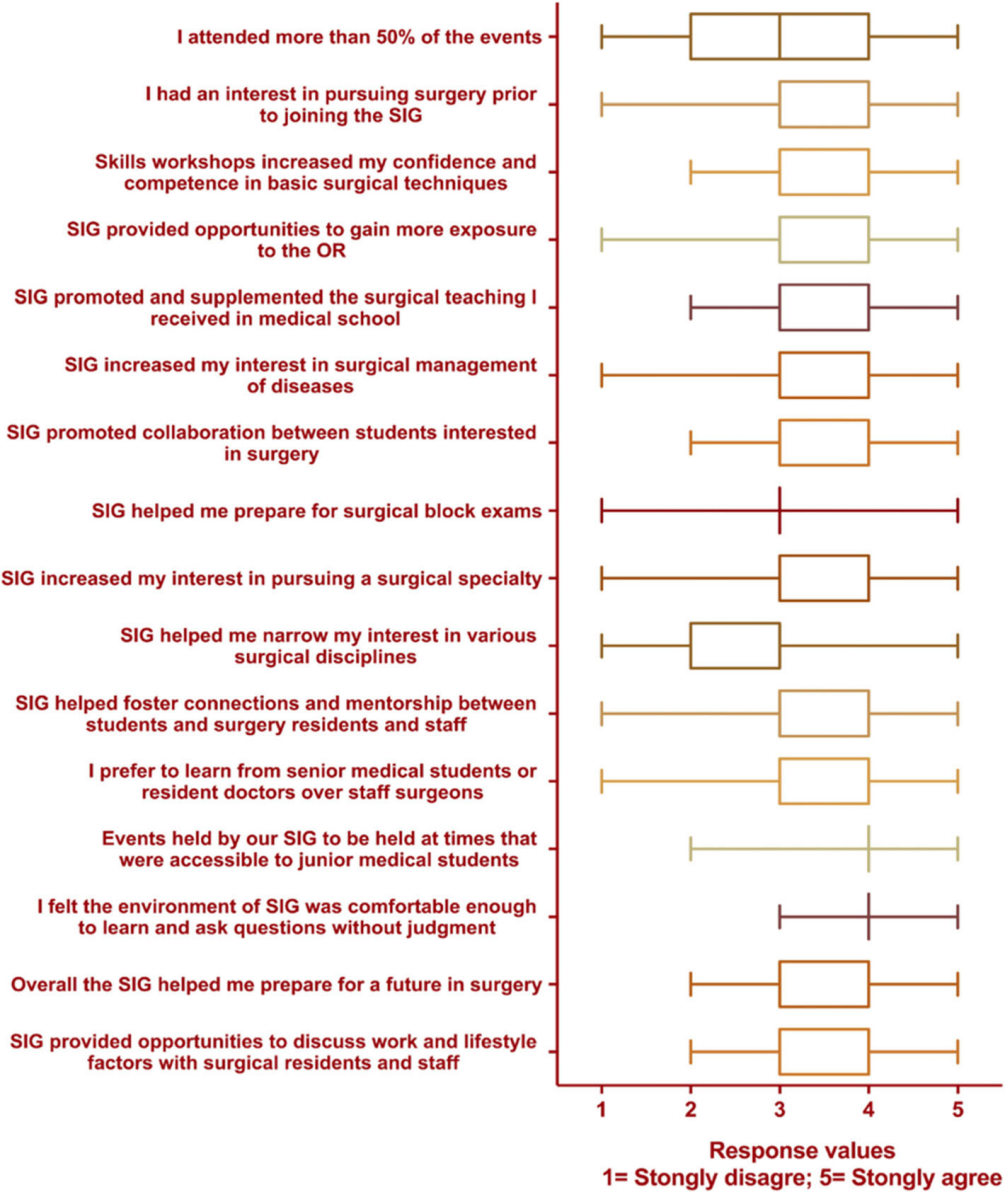
Box and whisker plots of survey responses from SIG members. Ratings are on an ordinal Likert scale, where 1 = Strongly disagree and 5 = Strongly agree. The box represents the interquartile range, and the whiskers the minimum and maximum values. Median values are displayed as separate lines in the box, but often overlap with interquartile values and are not displayed

A total of 127 members responded. The survey was distributed to an unknown number of SIG members, as each executive disseimated the survey using various methods that were not possible to track quantitatively. The majority of SIG members were females in their early-to-mid 20s and pre-clerkship stage of education (Table 4). On average, members felt they attended a majority (> 50%) of the events (Likert score of 3.32 ± 1.09), and had a prior interest in surgery (3.31 ± 1.09) before joining their surgical society (Fig. 1 & Table 5). Most members believed that workshops hosted by SIG increased their confidence and competence in basic surgical skills (3.89 ± 0.70), and helped them gain more exposure to the operating room (3.24 ± 0.94). They believed their SIG supplemented the surgical teaching they received in medical school well (3.62 ± 0.76), promoted collaboration (3.48 ± 0.71) and increase their interest in the surgical management of disease (3.51 ± 0.81). Most members, however, did not agree that SIG helped narrow their interests in various surgical disciplines (2.98 ± 0.80).

**Table 4:** Demographic characteristics of participating SIG members (*n* = 127)

Characteristics	Frequency	
	n	(%)
Overall	127	
Gender		
Female	82	65
Male	45	35
Age		
19–20	2	2
21–22	33	26
23–24	50	40
25–26	26	21
27–28	8	6
29–30	7	6
Year of study		
Med 1	66	52
Med 2	55	43
Med 3	3	2
Med 4	3	2

**Table 5:** Survey responses from SIG members

	Mean	SD
I attended more than 50% of the events	3.32	1.09
I had an interest in pursuing surgery prior to joining the SIG	3.31	1.09
Skills workshops increased my confidence and competence in basic surgical techniques	3.89	0.70
SIG provided opportunities to gain more exposure to the OR	3.25	0.94
SIG promoted and supplemented the surgical teaching I received in medical school	3.63	0.76
SIG increased my interest in surgical management of diseases	3.51	0.81
SIG promoted collaboration between students interested in surgery	3.48	0.71
SIG helped me prepare for surgical block exams	2.89	0.56
SIG increased my interest in pursuing a surgical specialty	3.50	0.79
SIG helped me narrow my interest in various surgical disciplines	2.98	0.80
SIG helped foster connections and mentorship between students and surgery residents and staff	3.39	0.83
I prefer to learn from senior medical students or resident doctors over staff surgeons	3.23	0.91
Events held by our SIG to be held at times that were accessible to junior medical students	4.05	0.60
I felt the environment of SIG was comfortable enough to learn and ask questions without judgment	4.02	0.60
Overall the SIG helped me prepare for a future in surgery	3.41	0.61
SIG provided opportunities to discuss work and lifestyle factors with surgical residents and staff	3.88	0.75

Members felt the learning provided complemented their education (3.63 ± 0.76), but did not specifically help prepare for their block exams (2.89 ± 0.56). With respect to the learning environment, most members agreed that the events were accessible (4.04 ± 0.60) and felt comfortable asking questions (4.02 ± 0.60). Overall, members indicated that SIG increased their interest in pursuing a surgical career (3.50 ± 0.79), helped foster connections and mentorship between students, surgery residents and staff surgeons (3.39 ± 0.83), and gave opportunities to discuss lifestyle and work life balance (3.88 ± 0.75). Members agreed that SIG helped prepare them for a future in surgery (3.41 ± 0.61).

Surgical skills nights such as suturing and knot tying (73%) were most frequently selected as the most helpful events (Table 2). The most common responses for beneficial future events included more skills events (29%), panel discussions/Q&A sessions (10%), and improved shadowing opportunities (10%) (Table 6).

**Table 6:** Future events that respondents indicated may be beneficial (*n* = 42)

Response	Frequency	
	n	(%)
Overall	42	
More skills events/ Greater event capacity	12	29
Panel discussion/ Q&A session	4	10
Improved shadowing opportunities	4	10
Career fair	3	7
More information about surgical subspecialty	3	7
OR preparation/ Tour	2	5
More relaxed social events	2	5
Myth busting about a career in surgery	2	5
Anatomy classes	1	2
Hospital based activities during the holidays (Eg. observerships)	1	2
Clerkship tips	1	2
Event with first year residents about matching	1	2
Analyzing surgical videos	1	2
Information about surgeon supply/ demand	1	2
“Meet the surgeons” night	1	2
“Speed dating event” with residents/ surgeons	1	2
Structured mentorship	1	2
Information about technology in surgery	1	2

## Discussion

SIGs have become a widely employed, and highly effective approach to fostering medical student interest in surgical disciplines as well as fortifying their confidence and competence during crucial pre-clerkship years [5, 6, 14]. Our results affirm previous reports that surgical societies have the ability to provide students with opportunities to increase competence in basic surgical techniques, avenues to interact with faculty, address queries and stereotypes, and overall help prepare for a future in all surgical specialties [8, 11, 12, 16].

Our SIG member respondents seem to match the demographics of those across the country as pre-clerkship students in their 20s, with a mix of both genders [2, 3, 9]. However, our results indicate that the respondents were predominantly females. If we consider this a surrogate marker for student body interest in surgery, our study contradicts notions of increased difficulty for female medical students to pursue surgical specialties [2, 4]. This may reflect diminishing notions of gender roles in medicine, with a proportional growing number of aspiring female surgeons, a phenomenon reported in recent publications [3, 9]. However, we recognize that response rate is not necessarily correlated with overall likelihood to pursue a surgical specialty.

Our results in both Table 2 and Table 6 illustrate the most beneficial events to SIG members support two key hypotheses. First, it shows that medical students are driven towards visual learning paradigms as surgical skills workshops were cited as the most useful events. Our results confirm those of Ologunde et al., who described skills workshops as the most popular initiatives [14]. Second, it showcases the importance of lifestyle career nights, as they provides the opportunity to debunk erroneous views surrounding surgical lifestyle [17].

Given the trend towards earlier selection of career choices, responsive early exposure to surgical disciplines becomes essential for informed decision making. Dolan-Evans and colleagues illustrated the importance of early intervention by showing that interest in surgery decreased from 33 to 12.3% in the first two years of medical school [3]. A study from Columbia University reported tripled entrance rates into general surgery programs from medical graduates following establishment of their surgical interest group [6]. The overall correlation between surgical exposure and heightened interest is illustrated in previous studies reporting a two-fold increase in most surgical careers following clerkship [18–20]. Conversely, beyond increasing matriculation rates, SIGs involvement may provide the necessary experience, exposure, and discussion with departmental representatives to help students “rule out” surgery and decrease attrition rates during residency [5, 21].

In terms of operational barriers, finance was listed as the most common obstacle. This suggests future aid to societies should begin with financial support, to increase the number and capacity of events. Similarly, conflicts with other interest groups for time was another commonly described obstacle. Hence, transparent communication with other interest groups in order to negotiate mutually acceptable schedules seems to be a high yield point for improvement.

Our results illustrate that on average most students agreed that involvement in SIG helped develop connections and mentorships, which is crucial in maintaining interest [5, 16, 22]. Berger and colleagues showed first and second year medical students with mentor experience in any specialty were four times more likely to pursue surgery by clerkship [16]. Additionally, positive pre-clerkship experiences with surgeons and surgery may enable students to enter their rotations with a more enlightened and excited mindset, while also providing a buffer against negative experiences and comments. Our results also illustrate that the SIG helped increase interest in pursuit of surgical specialties and surgical management of disease.

Our study is not without limitations. Firstly, Table 1 reflects a large proportion of our executive respondents were from our local site and not across North America. Although this may overshadow other results, given the high homogeneity in responses and consistency with previous studies we feel this does not negate our ability to deduce meaningful results. Second, we recognize that we were not able to correlate survey response with actual matriculation to surgical programs in residency. Third, as the majority of our respondents were in the first year of medical school at the time of survey distribution, they may have only had a limited exposure to their SIG. However, this enables us to capture early impressions of medical students, which is a key focus as most decisions surrounding career and budding interest are developed early. Lastly, as our sample size of participants is small relative to the national medical student population.

## Conclusion

Medical school surgical interest groups can play a unique role in providing medical students with early exposure to various surgical disciplines and information surrounding influential questions of lifestyle and work life balance. This is relevant in the face of a broadening medical school curriculum and decreasing surgical applicants. Our study highlights the inner workings and potential benefits of SIGs, while exposing the various barriers and struggles executives face in the changing landscape of medical education.

## Additional files

Additional file 1: Blank copy of The Undergraduate Medical Surgery Interest Group: Improving an existing model survey. (PDF 11 kb)

Additional file 2: Blank copy of The Undergraduate Medical Surgery Interest Group: Member Experience survey. (PDF 43 kb)

## Abbreviations

SIG: surgery interest group
