# Supplementary material for: Cross sectional analysis of student-led surgical societies in fostering medical student interest in Canada
Source: BMC Med Educ. 2019 Mar 8;19:77. doi: 10.1186/s12909-019-1502-5 (PMC6408764; doi:10.1186/s12909-019-1502-5)
Supplement: Supplementary file 1 — Blank copy of The Undergraduate Medical Surgery Interest Group: Improving an existing model survey. (PDF 11 kb) [file 12909_2019_1502_MOESM1_ESM.pdf]

The Undergraduate Medical Surgery Interest Group: Improving an existing model

Q1: What institution are you from?

Q2: How did you get involved in the surgery interest group?

- ◯ Friend/ classmate
- ◯ Introduction in formal didactic lecture setting
- ◯ Resident
- ◯ Interest group fair
- ◯ Staff
- ◯ Other (please specify)

Q3: What made you first join the surgery interest group?

Q4: How did you become appointed as SIG chair/ leader?

○ Appointed by previous chairs ○ Voted in ○ Other (please specify)

Q5: How many executive/leaders were there?

Q6: What were the roles of the executive/leaders?

Q7: On average, how many hours per week did you dedicate to SIG events?

Q8: On average, what was your yearly budget?

Q9: Did you believe you had sufficient funding to run your planned events?

○ Yes ○ No ○ Unsure

If no, please describe:

Q10: What type of events did you hold throughout the year? (Please select all that apply)

- ☐ Lecture series
- ☐ Surgeon Q&A/ lifestyle night
- ☐ Resident Q&A/ lifestyle night
- ☐ Residency matching information night
- ☐ Scrubbing into OR/ sterile technique session
- ☐ Surgical skills night (Eg. Suturing, knots, basic laproscopic skills)
- ☐ Career night
- ☐ Full day events (Eg. Surgery saturday)
- ☐ Other (Please describe)

Q11: Which event was the most successful/ best received?

- ○ Lecture series
- ○ Surgeon Q&A/ lifestyle night
- ○ Resident Q&A/ lifestyle night
- ○ Residency matching information night
- ○ Scrubbing into OR/ sterile technique session
- ○ Surgical skills night (Eg. Suturing, knots, basic laproscopic skills)
- ○ Career night
- ○ Full day events (Eg. Surgery saturday)
- ○ Other (Please describe)

Q12: Did you help facilitate any of the following programs?

- ◯ SEAD program (Surgery Education and Discovery)
- ◯ Transplant procurement pager program
- ◯ Mentorship programs (With residents)
- ◯ Women in surgery
- ◯ Observerships
- ◯ On-call shift night
- ◯ Mentorships programs (With staff)
- ◯ Other (Please specify)

Q13: The majority of events hosted by your interest group were run by which of the following:

- ◯ Senior Medical Students
- ◯ Residents
- ◯ Staff
- ◯ Others (Please specify)

Q14: On average, what proportion of anticipated students attended your events?

- ◯ 0-19%
- ◯ 20-39%
- ◯ 40-59%
- ◯ 60-79%
- ◯ 80-99%

Q15: Interest in your surgery interest group was maintained by students throughout the year

○ Strongly disagree ○ Disagree ○ Neutral ○ Agree ○ Strongly agree

Q16: Did you perform any of the following advertising or promotion of your interest group?

○ E-mails ○ Posters ○ Class announcements ○ Interest group fairs
○ Flyers ○ Other (Please specify)

Q17: Did you receive any of the following Faculty/ university support for your interest group

○ Financial ○ Administrative (Eg. helping organize events)
○ Promotional ○ Staff surgeon liaison
○ Other (Please specify)

Q18: What barriers did you face in your role as SIG chair? (Please select all that apply)

☐ Financial
☐ Trouble getting space for events
☐ Poor resident involvement
☐ Poor faculty involvement
☐ Poor departmental/ university involvemnet
☐ Poor student engagement
☐ Conflicts with other interest groups for time
☐ Trouble balancing medical school and the surgery interest group
☐ Other (Please describe)

Q19: Which barrier was the single biggest obstacle during your tenure as chair?

- ◯ Financial
- ◯ Trouble getting space for events
- ◯ Poor resident involvement
- ◯ Poor faculty involvement
- ◯ Poor departmental/ university involvemnet
- ◯ Poor student engagement
- ◯ Conflicts with other interest groups for time
- ◯ Trouble balancing medical school and the surgery interest group
- ◯ Other (Please describe)

Q20: How do you think your SIG could improve?

Q21: Would like to see a universal, Canadian surgery interest group be developed?

○ Yes ○ No ○ Unsure

If no, please describe
