# Supplementary material for: Cross sectional analysis of student-led surgical societies in fostering medical student interest in Canada
Source: BMC Med Educ. 2019 Mar 8;19:77. doi: 10.1186/s12909-019-1502-5 (PMC6408764; doi:10.1186/s12909-019-1502-5)
Supplement: Supplementary file 2 — Blank copy of The Undergraduate Medical Surgery Interest Group: Member Experience survey. (PDF 43 kb) [file 12909_2019_1502_MOESM2_ESM.pdf]

The Undergraduate Medical Surgery Interest Group: Member Experience

Q1: Please indicate your age

[ ]

Q2: Please indicate the gender you identify with

◯ Male ◯ Female ◯ Prefer not to say

Q3: When I was a member of the SIG, I attended more than 50% of the events

◯ Strongly disagree ◯ Diagree ◯ Neutral ◯ Agree ◯ Strongly Agree

Q4: I had an interest in pursuing surgery prior to joining the SIG

◯ Strongly disagree ◯ Diagree ◯ Neutral ◯ Agree ◯ Strongly Agree

Q5: The skills workshops increased my confidence and competence in basic surgical techniques (Eg. suturing, scrubbing in)

◯ Strongly disagree ◯ Diagree ◯ Neutral ◯ Agree ◯ Strongly Agree

Q6: The SIG provided opportunities to gain more exposure to the operating room

◯ Strongly disagree ◯ Diagree ◯ Neutral ◯ Agree ◯ Strongly Agree

Q7: The SIG promoted and supplemented the surgical teaching I received in medical school

◯ Strongly disagree ◯ Diagree ◯ Neutral ◯ Agree ◯ Strongly Agree

Q8: The SIG increased my interest in surgical management of diseases

◯ Strongly disagree ◯ Diagree ◯ Neutral ◯ Agree ◯ Strongly Agree

Q9: The SIG promoted collaboration between students interested in surgery

○ Strongly disagree ○ Diagree ○ Neutral ○ Agree ○ Strongly Agree

Q10: The SIG helped me prepare for surgical block exams

○ Strongly disagree ○ Diagree ○ Neutral ○ Agree ○ Strongly Agree

Q11: The SIG increased my interest in pursuing a surgical specialty

○ Strongly disagree ○ Diagree ○ Neutral ○ Agree ○ Strongly Agree

Q12: The SIG helped me narrow my interest in various surgical disciplines

○ Strongly disagree ○ Diagree ○ Neutral ○ Agree ○ Strongly Agree

Q13: The SIG helped foster connections and mentorship between students and surgery residents and staff

○ Strongly disagree ○ Diagree ○ Neutral ○ Agree ○ Strongly Agree

Q14: I prefer to learn from senior medical students or resident doctors over staff surgeons

○ Strongly disagree ○ Diagree ○ Neutral ○ Agree ○ Strongly Agree

Q15: I found the events held by our SIG to be held at times that were accessible to junior medical students

○ Strongly disagree ○ Diagree ○ Neutral ○ Agree ○ Strongly Agree

Q16: I felt the environment of SIG was comfortable enough to learn and ask questions without judgment

○ Strongly disagree ○ Diagree ○ Neutral ○ Agree ○ Strongly Agree

Q17: Overall the SIG helped me prepare for a future in surgery

○ Strongly disagree ○ Diagree ○ Neutral ○ Agree ○ Strongly Agree

Q18: The SIG provided opportunities to discuss work and lifestyle factors with surgical residents and staff

- ○ Strongly disagree
- ○ Diagree
- ○ Neutral
- ○ Agree
- ○ Strongly Agree

Q19: The SIG event that was most helpful was:

- ○ Lifestyle/ career nights
- ○ Skills nights (Eg. Suturing, knots, etc.)
- ○ Q/A with staff
- ○ Q/A with residents
- ○ Other (Please specify)

Q20: An event I think might be beneficial in the future:
